# Quercetin inhibits intestinal non-haem iron absorption by regulating iron metabolism genes in the tissues

**DOI:** 10.1007/s00394-018-1680-7

**Published:** 2018-03-28

**Authors:** Marija Lesjak, Sara Balesaria, Vernon Skinner, Edward S. Debnam, Surjit Kaila S. Srai

**Affiliations:** 1 Division of Biosciences, Research Department of Structural and Molecular Biology, University College London, Darwin Building, Gower Street, London, WC1E 6BT UK; 2 Department of Chemistry, Biochemistry and Environmental Protection, Faculty of Sciences, University of Novi Sad, Trg Dositeja Obradovića 3, Novi Sad, 21000 Serbia; 3 Division of Biosciences, Research Department of Neuroscience, Physiology and Pharmacology, University College London, Royal Free Campus, Rowland Hill Street, London, NW3 2PF UK

**Keywords:** Non-haem iron, Absorption, Duodenum, Quercetin, Polyphenols, Iron deficiency anaemia

## Abstract

**Purpose:**

There is general agreement that some dietary polyphenols block non-haem iron uptake, but the mechanisms by which they achieve this action are poorly understood. Since the polyphenol quercetin is ingested daily in significant amounts, we have investigated the effect of quercetin on duodenal non-haem iron absorption in vivo, as well as its effect on factors known to be involved in systemic iron metabolism.

**Methods:**

Rats were subject to gastric gavage and systemic quercetin administration. Treatments were followed with uptake studies using radiolabeled iron, serum iron and transferrin saturation measurements, LC-MS/MS analysis of quercetin metabolites in serum, determination of tissue non-haem iron content and analysis of gene expression of iron-related proteins.

**Results:**

Both oral and intraperitoneal (IP) quercetin caused serum and tissue iron depletion by two means, first by increasing mucosal iron uptake and inhibiting iron efflux from duodenal mucosa, and second by decreasing levels of duodenal DMT1, Dcytb and FPN. Additionally, IP quercetin induced highly significant increased liver expression of hepcidin, a hormone known to inhibit intestinal iron uptake.

**Conclusions:**

Oral quercetin significantly inhibited iron absorption, while IP quercetin significantly affected iron-related genes. These results could lead to development of new effective ways of preventing and treating iron deficiency anaemia, the most widespread nutritional disorder in the world.

**Electronic supplementary material:**

The online version of this article (10.1007/s00394-018-1680-7) contains supplementary material, which is available to authorized users.

## Introduction

Maintaining systemic iron levels within narrow limits is important for human health. Iron homeostasis is maintained by regulating the iron levels in plasma (transferrin-bound iron), which is maintained by four coordinated processes: duodenal iron absorption, macrophage iron recycling, hepatic iron storage and erythropoiesis [1].

Erythropoiesis requires nearly 30 mg iron each day, the main part of which comes from the recycling of iron via reticulo-endothelial macrophages (> 28 mg/day). Each day 1–2 mg of iron is absorbed by duodenal enterocytes and 1–2 mg of iron is lost by nonspecific obligatory processes. Since there is no known physiological mechanism for controlling iron excretion and that macrophage-mediated iron recycling cannot be sufficient for maintaining erythropoiesis over the long term, especially during periods of growth and pregnancy, efficient duodenal absorption of dietary iron is critical for body iron homeostasis [1, 2].

Average dietary iron bioavailability is low. Approximately 10–20 mg of iron is consumed daily, from which only 10% is absorbed. Non-haem iron is present in food from both animal and plant origin, whilst haem iron is present only in food of animal origin. Despite its abundance in the diet, the rate of non-haem iron absorption is much lower than that of haem iron [3].

Generally, the amount of absorbed iron fulfils daily needs, but it can be easily reduced eventually leading to iron deficiency anaemia (IDA). IDA is the most widespread nutritional disorder affecting more than 30% of the world’s population. The consequences of long-lasting IDA can be severe and contribute to 20% of all maternal deaths, poor pregnancy outcome, premature births, low birth weight babies, morbidity in infants, impaired physical and cognitive development of children and reduced work productivity in adults [4].

Non-haem iron is mainly present in the diet as Fe^3+^. But this must be reduced to the Fe^2+^ form to be available for absorption. This is achieved by the combined action of duodenal cytochrome B (Dcytb), a ferrireductase that is present on the apical membrane of duodenal enterocytes, or dietary reducing agents, such as ascorbic acid. Iron in the Fe^2+^ form is then transported across the apical duodenal membrane by the iron symporter, divalent metal transporter 1 (DMT1). Fe^2+^ is subsequently transferred to the blood via the iron exporter, ferroportin (FPN), reoxidised by a membrane-attached ferroxidase, hephaestin, and bound to plasma transferrin [5].

FPN is not only an iron exporter, but it is also a receptor for hepcidin, a peptide which is particularly important during iron overload [2]. As a result of hepcidin binding to FPN, endocytosis of FPN occurs, leading to its proteolysis in lysosomes. Hepcidin is the main systemic regulator of iron homeostasis since it controls iron efflux from cells, thereby maintaining circulating iron at a level that prevents iron overload [6]. By binding to FPN, hepcidin controls the main supply routes of iron into the circulation, such as iron absorbed from the diet in duodenal enterocytes, iron from macrophages liberated during recycling of senescent erythrocytes and iron which is stored in hepatocytes [6].

It is generally believed that bioavailability of non-haem iron highly depends on the presence of dietary promoters or inhibitors of iron absorption. Among inhibitors of non-haem iron absorption, particular dietary polyphenols are considered to be extremely potent [7]. Polyphenols are a group of plant secondary metabolites which are present in nearly all food and beverages of plant origin. Significant amounts of polyphenols are consumed daily, approximately 1 g, and these compounds are receiving increasing attention worldwide due to their proven health benefits [8]. However, the negative impact of some polyphenols on non-haem iron absorption has been highlighted previously [7, 9–12]. The exact mechanism by which certain polyphenols reduce bioavailability of non-haem iron is not fully understood, but it is proposed that polyphenols are able to chelate non-haem iron [7, 13, 14]. In marked contrast, work using Caco-2 cells showed that certain polyphenols promote iron bioavailability (i.e. epicatechin, kaempferol [15, 16]).

It has also been shown that different polyphenols can alter hepatic hepcidin expression in vivo which provides an additional mechanism for the role of polyphenols in iron metabolism [17, 19‒19] and indicates their potential therapeutic use in disorders of iron metabolism. However, published results indicate that some dietary polyphenols strongly increase hepcidin expression (i.e. quercetin, genistein), whilst others have opposite action (i.e. myricetin). Thus, more work is necessarily to make conclusions concerning the precise role of dietary polyphenols in iron metabolism, particularly their nutritional importance in maximizing iron bioavailability.

The polyphenol quercetin, a well-known iron chelating agent [20], is ingested daily in great amounts (16 mg/day; [21]). As an example, it is estimated that red onion, common onion, cranberry, blueberry and fig have 39, 20, 15, 8 and 5 mg of quercetin aglycone per 100 g of fresh weight of edible portion, respectively [22]. It was therefore considered worthwhile to further investigate the effect of quercetin on duodenal non-haem iron absorption in vivo as well as its effect on systemic iron metabolism.

## Materials and methods

### Materials

Low iron diet was obtained by Special Diet Service, UK. Pentobarbitone sodium (Pentoject) was obtained from Animalcare Ltd., UK. ^59^Fe was obtained by PerkinElmer, USA. Quercetin-3,4′-di-*O*-glucoside, quercetin-3-*O*-glucuronide, isorhamnetin-3-*O*-glucoside and isorhamnetin were obtained by Extrasynthese, France. All other chemicals were purchased from Sigma-Aldrich, USA.

### Animal care and treatments

Experiments used male Sprague–Dawley (SD) rats supplied by the Comparative Biology Unit, Royal Free Campus, University College London (UCL), London, UK. All procedures were approved by the UCL local animal ethics committee and were conducted in accordance with the UK Animals (Scientific Procedures) Act, 1986, and have therefore been performed in accordance with the ethical standards laid down in the 1964 Declaration of Helsinki and its later amendments. After weaning (3 weeks old) rats were placed on a low iron diet (25 µg of iron/g dry food) for 2 weeks ad libitum and allowed free access to water throughout. Subsequently, animals were subject to different gavage and intraperitoneal injection (IP) treatments with quercetin (50 mg/kg) and DMSO (10% v/v). Five animals were used per group.

Short-term treatments comprised two experimental regimes. First, single gavage or IP treatments were given 5 h prior to uptake studies (gavage and IP) or the removal of tissues (only IP). In addition, two gavage or IP treatments were carried out, the first one 18 h and second one 5 h prior to uptake studies (gavage and IP) or the removal of tissues (only IP).

For long-term studies, quercetin and DMSO gavage treatments were applied daily for 10 days. On the tenth day, gavage treatment was performed 4 h prior to the removal of tissues.

The quercetin dose used (50 mg/kg) was calculated according to Reagan-Shaw et al. [23]. The human equivalent dose for the rat is 8 mg/kg, which corresponds to around 500 mg of quercetin consumed daily by adults. The daily recommended quercetin supplement dose is 200–1200 mg, which is also in accordance with the quercetin dose applied in our study [24].

Following administration of a terminal IP dose of pentobarbitone sodium (120 mg/kg body weight), a blood sample was removed via cardiac puncture. Serum was separated from the clotted blood after centrifugation (10 min at 5000*g*), rapidly frozen in liquid nitrogen before being stored at − 80 °C and later used for serum iron and transferrin saturation measurements, as well as LC-MS/MS analysis of quercetin metabolites. Additionally, duodenum, liver and spleen were removed and rapidly frozen in liquid nitrogen before being stored at − 80 °C, and subsequently used for determination of tissue non-haem iron content and gene expression levels.

### In vivo iron uptake study

After the animal care procedure and short-term treatments, four groups (two gavage and two IP) of rats were subjected to uptake studies. Animals were anesthetized with pentobarbitone sodium (60 mg/kg body weight, IP) and 10 cm of duodenum (starting 1 cm distal to the pylorus) was cannulated and rinsed free of its contents with warm saline (0.9% w/v of NaCl), followed by air. Uptake buffer (200 µL), containing Na-HEPES (14.6 mmol/L), NaCl (127.4 mmol/L), KCl (3.2 mmol/L), ascorbic acid (4.0 mmol/L) and ^59^Fe^2+^ (0.2 mmol/L) was instilled into the duodenal segment, which was then tied off. Body temperature was maintained at 37 °C using a heating blanket. After 30 min, blood samples (≤ 2 mL) were collected via cardiac puncture and put in pre-weighed tubes, allowing blood weight to be determined. The cannulated segment of duodenum was removed and washed with approximately 40 mL of solution containing NaCl (154 mmol/L), ascorbic acid (0.1 mmol/L) and FeCl_3_ (0.01 mmol/L) to displace any ^59^Fe bound to the mucosal surface. The segment was cut longitudinally to form a flat sheet, and the mucosa was removed by scraping, placed into a pre-weighed tube, and its weight determined. Appropriate blood and mucosa samples were gamma counted (Wallac 1282 Compugamma Counter Model 1283) for the determination of ^59^Fe activity, representing ^59^Fe level of absorbed and sequestered in the mucosa, respectively. Results were expressed as a percentage of absorbed ^59^Fe retained in duodenal mucosa or transferred to blood. Total absorbed radioactive iron was taken to be that distributed between mucosa of cannulated duodenum and total body blood. The percentage of ^59^Fe transferred to the entire blood volume of the animal was calculated using the equation: total blood volume (mL) = body weight (g) × 0.06 + 0.77.

### Serum iron and transferrin saturation measurements

Serum iron and transferrin saturation were measured using a commercial kit according to manufacturer’s instructions (Pointe Scientific Inc., USA).

### LC-MS/MS analysis of quercetin and selected quercetin metabolites in rat serum

LC-MS/MS analysis of serum was used to quantify levels of quercetin and selected quercetin metabolites (quercetin-3,4′-di-*O*-glucoside, quercetin-3-*O*-glucuronide, isorhamnetin-3-*O*-glucoside and isorhamnetin). To remove proteins from serum prior to LC-MS/MS analysis, acetone (50 µL) and CH_3_COOH (2 µL) were vigorously mixed with serum (50 µL). Additionally, 1 µL genistein (1 µg/mL in methanol) was added to mixture as internal standard. Mixture was centrifuged (15 min at 7000*g*), supernatant was removed and subjected to further LC-MS/MS analysis.

LC-MS/MS analysis was performed on an Agilent Technologies 1200 Series high-performance liquid chromatograph coupled with Agilent Technologies 6410A Triple Quad tandem mass spectrometer with electrospray ion source, and controlled by Agilent Technologies MassHunter Workstation software—Data Acquisition (ver. B.03.01). Separation was carried out using a Zorbax Eclipse XDB-C_18_ analytical column (4.6 × 50 mm, 1.8 µm particle size). The column was maintained at 50 °C and a binary gradient separation was performed using a flow rate of 1 mL/min. The mobile phase is consisted of 0.05% HCOOH in water (A) and 100% methanol (B). The gradient profile was starting with 30% B, reaching 70% B in 6 min, then 100% B at 9 min, holding 100% B until 12 min, with post-time of 3 min. The injection volume was 10 µL and the autosampler needle was washed with acetonitrile between injections to eliminate carryover. Eluted components were detected by MS, using the ion source parameters as follows: nebulization gas (N_2_) pressure 60 psi, drying gas (N_2_) flow 11 L/min and temperature 350 °C, capillary voltage 4 kV. All compounds were quantified in selected reaction monitoring mode. Compound-specific, optimized MS/MS parameters are given in Supplementary material Table 1. For all the compounds, peak areas were determined using Agilent MassHunter Workstation Software—Qualitative Analysis (ver. B.03.01). Calibration curves were plotted and sample concentrations calculated using the OriginLabs Origin Pro (ver. 8.0) software.

### Tissue non-haem iron determination

Tissues (duodenum, liver, spleen) were dried at 50 °C for 72 h and subsequently weighed. Quantitative of non-haem iron was carried out according to the method of Torrance and Bothwell [25]. Results were calculated as micrograms iron/gram of dry tissue weight (dw).

### RNA extraction and RT-PCR

Total RNA from tissues (duodenum, liver, spleen) was extracted with TRIzol^®^ reagent (Thermo Fisher Scientific) according to the manufacturer’s instructions. The concentration of extracted RNA was measured using a NanoDrop 2000c UV–VIS spectrophotometer (Thermo Scientific). Total RNA (1 µg) was reverse transcribed using the Verso cDNA reverse transcription kit (Thermo Fisher Scientific) according to the manufacturer’s instructions.

RT-PCR reactions were performed using a LightCycler^®^ 480 System (Roche Diagnostics GmbH, Germany) and a LightCycler^®^ 480 SYBR Green I Master kit (Roche Diagnostics) using GAPDH as internal standard. Each reaction was performed in duplicate and contained 0.3 µmol/L of each specific primer, forward and reverse, 6 µL of SYBR Green I Master, 1 µL of cDNA made up by diethyl pyrocarbonate treated water to 11 µL for each sample. Samples without cDNA were included as negative controls. Primers for genes of interest were synthesized by Sigma-Aldrich. Primers for the internal standard gene GAPDH were obtained from Primer Design, UK. The primer sequenced used was as follows: Dcytb forward, 5′-TCCTGAGAGCGATTGTGTTG-3′ and reverse, 5′-TTAATGGGGCATAGCCAGAG-3′; DMT1 forward, 5′-GCTGAGCGAAGATACCAGCG-3′ and reverse, 5′-TGTGCAACGGCACATACTTG-3′; FPN forward, 5′-TTCCGCACTTTTCGAGATGG-3′ and reverse, 5′-TACAGTCGAAGCCCAGGAC-3′; hepcidin forward, 5′-AGACACCAACTTCCCCATATG-3′ and reverse, 5′-ACAGAGACCACAGGAGGAATTCT-3′. Cycle threshold (Ct) values were obtained for each gene of interest and the GAPDH internal standard. Gene expression was normalized to GAPDH and represented as ΔCt values. For each sample, the mean of the ΔCt values was calculated. Relative gene expression was normalized to 1.0 (100%) of controls.

### Statistics

All data are presented as mean ± standard error of the mean (SEM). Statistical significant difference between control and quercetin treatment groups was determined using the Student’s two-tailed unpaired *t* test. Statistical significance was taken to be *p* ≤ 0.05.

## Results

### Short-term oral quercetin decreases, while IP quercetin has no effect, on non-haem iron absorption in duodenum

**Fig. 1:**
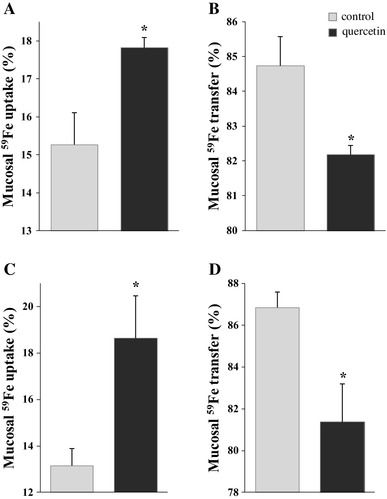
Short-term effect of orally administered quercetin on iron absorption in duodenum in rats. Rats were given a single or double gavage-containing quercetin or DMSO (control). Single quercetin treatment was applied 5 h before mucosal ^59^Fe uptake (**a**) and ^59^Fe transfer (**b**) was measured. Double quercetin treatments were applied 18 and then 5 h before mucosal ^59^Fe uptake (**c**) and ^59^Fe transfer (**d**) were measured. Data are mean ± SEM; *n* = 5 rats per group; *denotes significant difference from the control group (*p* ≤ 0.05)

The main aim of the gavage experiments was to investigate whether intake of quercetin influences non-haem iron absorption in the duodenum 5 or 18 h after administration of the polyphenol. Results showed that mucosal ^59^Fe uptake was significantly increased 5 h after a single gavage of quercetin given orally by gavage (Fig. 1a). In contrast, in these animals, mucosal ^59^Fe transfer to circulation was significantly decreased after the same treatment (Fig. 1b). For animals twice-gavaged with quercetin, 18 and then 5 h before the uptake experiment, the effect on iron absorption was even more pronounced compared with that seen after single gavage. Thus, mucosal ^59^Fe uptake was significantly increased, from 13 to 18% (Fig. 1c), whilst mucosal ^59^Fe transfer was significantly decreased, from 87 to 82% (Fig. 1d).

**Fig. 2:**
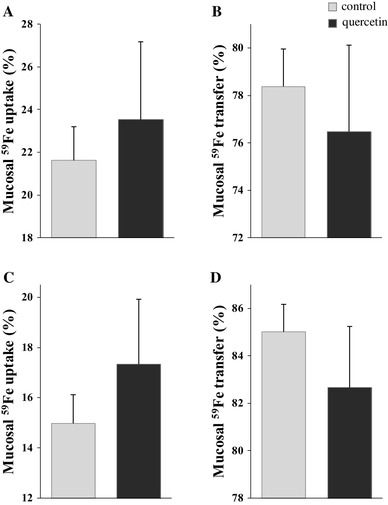
Short-term effect of IP-administered quercetin on iron absorption in duodenum in rats. Rats were given a single or double IP-containing quercetin or DMSO (control). Single quercetin treatment was applied 5 h before mucosal ^59^Fe uptake (**a**) and ^59^Fe transfer (**b**) was measured. Double quercetin treatments were applied eighteen and then 5 h before mucosal ^59^Fe uptake (**c**) and ^59^Fe transfer (**d**) were measured. Data are mean ± SEM; *n* = 5 rats per group

Uptake experiments after IP quercetin treatments were carried out to examine whether systemic short-term administration influence iron absorption. However, after a single or double IP quercetin treatment, mucosal ^59^Fe uptake and transfer remained the same (Fig. 2a–d).

### Long-term oral quercetin reduces tissue iron pools and expression of non-haem iron transporters in enterocytes

**Fig. 3:**
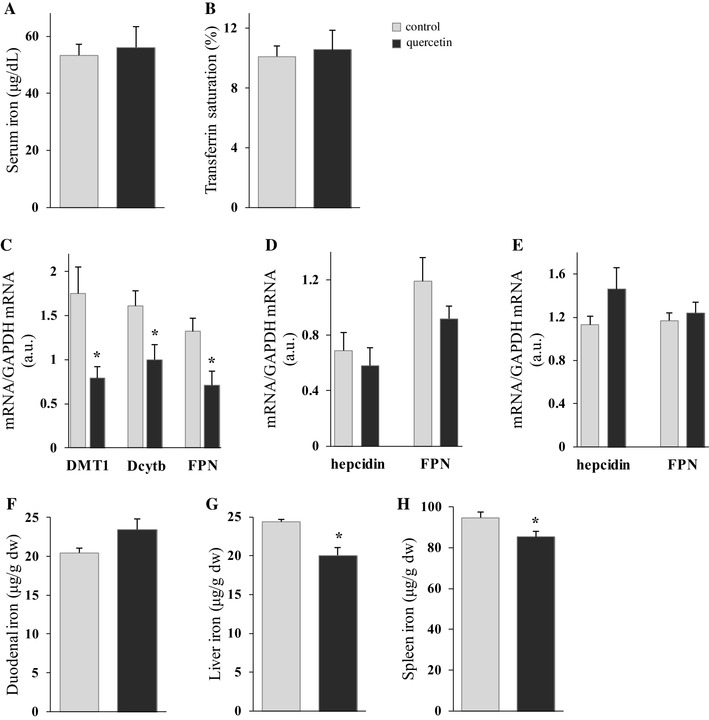
Long-term effect of orally administered quercetin on serum iron and transferrin saturation levels, iron-related gene expression in duodenum, liver and spleen, and duodenal, liver and spleen iron content in rats. The long-term effect of quercetin on serum iron (**a**) and transferrin saturation (**b**), iron-related gene expression in duodenum (**c**), liver (**d**) and spleen (**e**), and duodenal (**f**), liver (**g**) and spleen iron content (**h**) were measured after oral administration of quercetin or DMSO (control), during 10 days, single per day. Data are mean ± SEM; *n* = 5 rats per group; *denotes significant difference from the control group (*p* ≤ 0.05)

The long-term effect of quercetin on parameters of iron metabolism was studied after daily oral administration of quercetin or DMSO over a 10 day period. The aim was to ascertain whether long-term intake of quercetin influences non-haem iron absorption. This protocol was designed to emulate dietary regimes in man where ingested food is rich in quercetin. Surprisingly, serum iron and transferrin saturation levels were found to be unchanged after 10 days of oral quercetin treatment (Fig. 3a, b). However, duodenal gene expression of DMT1, FPN and Dcytb mRNA of rats gavaged with quercetin for 10 days showed a significant decrease in all mRNA investigated (2.2-, 1.8- and 1.6-fold, respectively; Fig. 3c). Analysis of hepcidin and FPN mRNA expression in liver and spleen showed no difference in related mRNA levels (Fig. 3d, e) between treated and control animal groups. In addition, after long-term oral quercetin liver and spleen iron levels decreased significantly (Fig. 3g, h), whilst there was no change in duodenal iron levels (Fig. 3f).

### Intraperitoneal treatment with quercetin induces iron depletion by increasing hepcidin and decreasing non-haem iron transporters expression

**Fig. 4:**
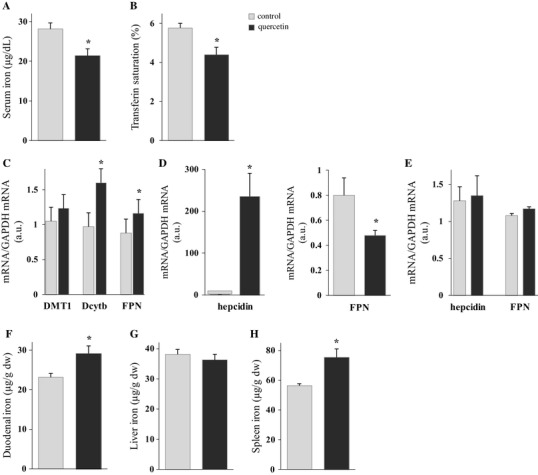
Short-term effect of single IP quercetin on serum iron and transferrin saturation levels, iron-related gene expression in duodenum, liver and spleen, and duodenal, liver and spleen iron content in rats. The short-term effect of quercetin on serum iron (**a**) and transferrin saturation (**b**), iron-related gene expression in duodenum (**c**), liver (**d**) and spleen (**e**), and duodenal (**f**), liver (**g**) and spleen iron content (**h**) were measured after single IP of quercetin or DMSO (control) 5 h before dissection. Data are mean ± SEM; *n* = 5 rats per group; *denotes significant difference from the control group (*p* ≤ 0.05)

**Fig. 5:**
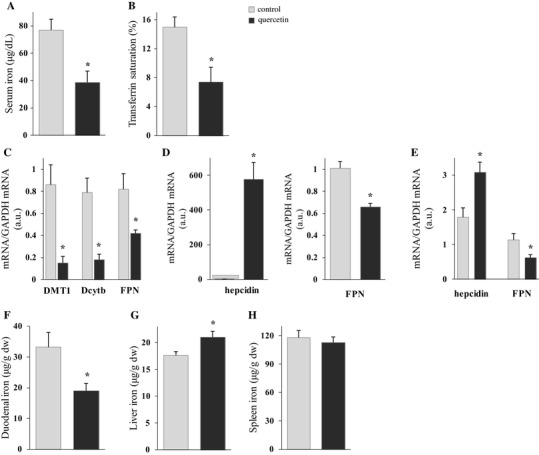
Short-term effect of double IP quercetin on serum iron and transferrin saturation levels, iron-related gene expression in duodenum, liver and spleen, and duodenal, liver and spleen iron content in rats. The short-term effect of quercetin on serum iron (**a**) and transferrin saturation (**b**), iron-related gene expression in duodenum (**c**), liver (**d**) and spleen (**e**), and duodenal (**f**), liver (**g**) and spleen iron content (**h**) were measured after double IP of quercetin or DMSO (control) 18 and then 5 h before dissection. Data are mean ± SEM; *n* = 5 rats per group; *denotes significant difference from the control group (*p* ≤ 0.05)

The effect of IP quercetin on iron metabolism was studied in rats after single IP (5 h; all results shown in Fig. 4) or double IP (18 and then 5 h; all results shown in Fig. 5) treatments. These experiments were designed to examine whether systemic administration of quercetin influences iron absorption and parameters of body iron status. Results showed that serum iron and transferrin saturation levels were significantly decreased after both single and double administration of quercetin (Figs. 4a, b, 5a, b, respectively). Surprisingly, Dcytb and FPN mRNA levels increased significantly after single treatment, 1.6- and 1.3-fold, respectively (Fig. 4c), whilst after double quercetin IP dose, analysis of the same mRNA in duodenum showed that DMT1, Dcytb and FPN mRNA levels were all significantly decreased, 5.7-, 4.4- and 2.0-fold, respectively (Fig. 5c). Liver hepcidin mRNA was significantly up-regulated after both single and double quercetin administration: 175- and 1031-fold, respectively (Figs. 4d, 5d, respectively). This was accompanied by a significant decrease in FPN mRNA levels in liver (Figs. 4d, 5d, respectively). Levels of splenic hepcidin and FPN mRNA did not change after single IP administration (Fig. 4e), but after double IP treatment hepcidin mRNA increased significantly whilst FPN decreased significantly (Fig. 5e). Furthermore, duodenal and spleen iron levels increased after single IP injection, while after double quercetin IP, duodenal iron level decreased and liver iron increased (Figs. 4f, h, 5f, g, respectively).

### Quercetin metabolites were present in serum only after IP quercetin administration

Quantitative analyses of quercetin and four selected quercetin metabolites in rat serum after short- and long-term quercetin treatments were performed using the LC-MS/MS technique. LC-MS/MS analysis was applied to give an overview on what compounds are present in the circulation after quercetin treatments. None of the metabolites or parental quercetin was detected in the control samples. In contrast, quercetin-3-*O*-glucuronide (single IP 27.8 ± 1.78 ng/mL serum; double IP 22.6 ± 0.94 ng/mL serum), quercetin (single IP 0.48 ± 0.01 ng/mL serum; double IP 20.9 ± 0.65 ng/mL serum), and isorhamnetin (double IP 3.43 ± 0.2 ng/mL serum), were detected in the serum of rats treated systemically with quercetin, whereas none of the examined compounds was detected in serum after long-term oral treatment.

## Discussion

There is general acceptance of the negative impact of foodstuffs rich in certain polyphenols on non-haem iron bioavailability in vivo. Available data are mainly based on results of isotope absorption studies [7], but there is little information on the precise mechanisms by which specific dietary polyphenols affect non-haem iron absorption in vivo. Our previous study [14] revealed the mechanism of inhibition of non-haem iron absorption by quercetin. We showed that quercetin chelates iron and thus prevents its transport across the enterocyte. Increased apical iron uptake is accompanied by decreased iron release into the blood. However, Lesjak et al. [14] used a protocol where iron and quercetin were introduced into the duodenal lumen together. Proof of an effect of quercetin ingestion on iron absorption is however still missing. The gavage protocol used in the present study was designed to imitate the diet of individuals consuming quercetin both randomly and regularly. Results from our present study indicate that oral quercetin had significant effects on both iron absorption and gene expression of duodenal proteins involved in iron metabolism, as well as iron pools in liver and spleen. Specifically, during short- and long-term settings, oral quercetin generally caused iron depletion and this was evident from the significantly reduced absorption rate of non-haem iron, reduced liver and spleen iron pools and levels of duodenal iron transporters.

Uptake studies after short-term single and double quercetin gavage treatments significantly increased of mucosal iron uptake, while there was a notable decrease in iron efflux from enterocytes. For the first time, our results show that orally applied quercetin could decrease iron absorption by inhibiting its basolateral exit. Decreased enterocyte iron transfer might be due to intracellular chelation of iron by quercetin which increases apical uptake of iron, but prevents basolateral exit [14]. However, 18 or 5 h are long periods for quercetin to remain in the lumen or inside the enterocyte and be available to chelate iron due to its fast metabolism [26]. It is certainly possible that towards the end of a 5 h period, levels of quercetin are too low to form an effective complex with iron. In this scenario, the most probable explanation for its effect on iron absorption is to cause changes in the expression of proteins involved in iron metabolism. Our previous study showed that quercetin can mediate knockdown of FPN1 in Caco-2 cells post-transcriptionally, which could be an additional mechanism allowing quercetin to affect efflux of iron from enterocyte in vivo [14]. The possibility that quercetin, or its metabolites, also have direct effects on the expression of other proteins involved in iron absorption and systemic iron balance, and that together with chelation this action modifies iron metabolism, should be further considered.

Long-term (10 days) gavage quercetin treatment reduced mRNA levels of duodenal DMT1, Dcytb and FPN, which could be a consequence of a direct effect of quercetin or its metabolites. Even though examined quercetin metabolites were not detected in the serum of treated rats, there is a possibility that other quercetin metabolites affect the expression of iron-related genes.

In our study, oral quercetin did not cause a change in liver and spleen hepcidin mRNA expression. These results contrast with the data of Tang et al. [27], who showed in vivo that oral quercetin efficiently supports hepcidin expression by intensification of the BMP6/SMAD4 signalling pathway. In Tang et al. [27], up-regulation of liver hepcidin, on both protein and mRNA levels, was documented after 15 weeks oral treatment with quercetin (100 mg/kg). These conflicting results might reflect the different periods of quercetin treatment.

Furthermore, after a 10 days treatment regime with oral quercetin, animals became iron depleted, as both spleen and liver iron pools decreased. Interestingly, Zhang et al. [28] showed that quercetin reduced liver iron content after an induced tissue iron overload. This effect was attributed to the ability of quercetin to combine with non-haem iron in tissue, transport it to the bloodstream and excrete it from the body. We also confirmed this in the study for both liver and spleen, which strongly supports the need for further research on different polyphenols as potential new iron chelating drugs.

In addition to observing the effects of oral quercetin on iron uptake and transport, we wanted to inspect the possible therapeutic effect of quercetin on body iron overload. Our data indicate that IP quercetin had a significant effect on systemic iron metabolism by indirectly affecting iron absorption. We shown that IP quercetin induced iron depletion as indicated by significant reductions in serum iron and transferrin saturation levels, as well as an increased hepcidin mRNA and decreased expression of genes involved in duodenal iron absorption.

There are only two reports in the literature that reported a similar effect of quercetin on hepcidin expression in liver. Bayele et al. [18] reported that IP quercetin increased hepcidin expression after 18 h, which might involve the Nrf2 pathway. Bayele et al. [18] also showed that hepcidin induction by quercetin correlated with changes in serum iron levels and transferrin saturation, as well as with reduction in FPN mRNA, which is also in accordance with our present study. Vanhees et al. [29] showed that prenatal exposure to quercetin caused hepcidin induction in adult mice and the authors hypothesized that after birth, when pups were no longer exposed to quercetin, improved bioavailability of dietary iron sensed as body iron overload.

Our study shows for the first time that IP quercetin could increase hepcidin levels in both liver and spleen. Increased levels of hepcidin are expected to be followed by reduced FPN levels. This “seesaw” relationship between hepcidin and FPN expression is well-known [6] and our results are in agreement with this notion. After single and double IP quercetin treatment, levels of liver FPN mRNA decreased significantly. This action on FPN, which is believed to be indirectly driven by hepcidin induction, was more pronounced after double quercetin treatment, where FPN mRNA levels declined in both liver and spleen. High hepcidin, as well as the expected reduction in FPN levels, should be accompanied by increased retention of iron in liver and spleen [6]. This pattern was evidenced in the present study.

There is a general agreement that increased hepcidin levels decrease intestinal iron absorption [30, 31]. It is proven that hepcidin expression is inversely proportional to expression of intestinal DMT1, Dcytb and FPN expression in vivo [32, 35‒35]. However, this relationship was only partially confirmed in this study. Namely, after single quercetin IP levels of duodenal mRNA DMT1 stayed the same, whilst the levels of Dcytb and FPN were raised. These results were somewhat surprising particularly in the light of detected increased levels of hepcidin and duodenal iron. It would be expected that the levels of DMT1 were down-regulated by the post-transcriptional IRE/IRP control process, such that when iron levels are high, expression of DMT1 is suppressed. Still, FPN mRNA in duodenum could be also partially under IRE/IRP control, which when iron levels in tissue are high, expression of FPN is up-regulated [36]. However, after double IP quercetin treatment, duodenal mRNA levels of DMT1, Dcytb and FPN were significantly down-regulated and lower levels of duodenal iron were recorded.

Hepcidin up-regulation is followed by a reduction in serum iron and transferrin saturation [37]. This principle was confirmed in the present study since single and double quercetin IP reduced serum iron and transferrin saturation levels.

Surprisingly, IP quercetin treatment did not affect mucosal iron uptake or transfer since a double dose of quercetin up-regulated mRNA hepcidin levels whilst reducing intestinal iron transporter expression. Further studies need to be performed to confirm the actions of IP quercetin on iron absorption in vivo.

After IP quercetin administration, quercetin-3-*O*-glucuronide, quercetin and isorhamnetin were detected with LC-MS/MS in serum, whilst the same analysis did not detect these compounds after long-term oral treatment. This confirms previously known facts that quercetin absorbed in the intestine has a short half-life [26] and that one of the dominant quercetin metabolites in serum is quercetin glucuronide [38]. Bioavailability of quercetin, defined as the portion of an initially administered dose that reaches the systemic circulation unchanged, is very low, mostly due to its extensive metabolism [26]. Quercetin is present in plants mainly in its highly hydrophilic glycosylated forms, primarily as β-glycosides of various sugars which are more bioavailable in humans [39]. There are two main routes for absorption of quercetin glycosides by enterocytes. First, absorption goes via a transporter, followed by deglycosylation within the enterocyte by cytosolic glycosidase. Second, deglycosylation can occur by luminal hydrolases, followed by transport of the aglycone into enterocyte by passive diffusion or transporter-mediated. After absorption, further biotransformation of quercetin aglycone involves glucuronidation, sulfation and methylation of hydroxyl groups, which primarily occurs in enterocytes and hepatocytes [24]. However, by both ways quercetin ends up in the enterocytes as aglycone, which is then able to chelate iron or have direct effect on transcription on iron-related genes. Thus, in this study quercetin aglycone was used instead of quercetin glycoside. However, the possibility that quercetin metabolites could also be involved in the observed effects on iron uptake and metabolism should not be overlooked.

It is evident that different routes of quercetin application affect iron metabolism in different ways. Thus, IP treatment mainly affected systemic iron homeostasis, mostly by regulating hepcidin expression and indirectly causing reduction in iron absorption, while oral quercetin directly influenced iron absorption.

Interestingly, previous results of others indicate contradictory results on how different dietary polyphenols affect iron homeostasis. Thus, Mu et al. [19] reported that the polyphenol myricetin inhibits hepcidin expression induction in vivo by the BMP/SMAD signalling pathway. Quercetin and myricetin are very similar in structure, with myricetin having an extra hydroxyl group. The differences in effect of these two similar polyphenols on iron absorption indicate the complexity of responses to polyphenols. Furthermore, Zhen et al. [17] and Patchen et al. [40] showed that genistein, a main polyphenol from soya, and ipriflavone, synthetic analog derived from abundant dietary polyphenol daidzein, respectively, both strongly promote hepcidin expression in vivo. Recent studies by Grillo et al. [41] and Zhang et al. [42] indicate that natural products could have a major role in iron metabolism and may have potential in therapy of iron metabolism disorders.

In conclusion, oral and IP treatment with quercetin caused serum and tissue iron depletion in rats by two means—inhibition of iron absorption and altered expression of iron-related genes. Our data confirm that quercetin increases mucosal iron uptake and inhibits iron efflux from duodenal mucosa. Also, oral quercetin treatment decreased mRNA levels of duodenal DMT1, Dcytb and FPN, while IP quercetin induced hepcidin expression, both in liver and spleen. These results also indicate that oral quercetin has a significant inhibitory effect on iron absorption in duodenum, while IP quercetin significantly affects systemic iron regulation leading to iron depletion. However, the precise mechanism of the action of quercetin on iron metabolism remains to be defined.

Our study is important in that it could lead to development of new approaches to preventing and treating IDA, as well as alleviating the effects of clinical iron overload. Polyphenols might prove to be a novel therapy for diseases of body iron metabolism.

## Electronic supplementary material

Below is the link to the electronic supplementary material.

Supplementary material 1 (DOC 37 KB)

## References

[1] Hentze MW, Muckenthaler MU, Galy B, Camaschella C (2010). Two to tango: regulation of Mammalian iron metabolism. Cell.

[2] Ganz T, Nemeth E (2012). Hepcidin and iron homeostasis. Biochim Biophys Acta.

[3] Carpenter CE, Mahoney AW (1992). Contributions of heme and nonheme iron to human nutrition. Crit Rev Food Sci Nutr.

[4] WHO (2008). Worldwide prevalence of anaemia 1993–2005. WHO global database on anaemia.

[5] Pantopoulos K, Porwal SK, Tartakoff A, Devireddy L (2012). Mechanisms of mammalian iron homeostasis. Biochemistry.

[6] Nemeth E, Tuttle MS, Powelson J, Vaughn MB, Donovan A, Ward DM, Ganz T, Kaplan J (2004). Hepcidin regulates cellular iron efflux by binding to ferroportin and inducing its internalization. Science.

[7] Petry N, Watson RR, Preedy VR, Zibadi S (2014). Polyphenols and low iron bioavailability. Polyphenols in human health and disease.

[8] Havsteen BH (2002). The biochemistry and medical significance of the flavonoids. Pharmacol Ther.

[9] Cook JD, Reddy MB, Hurrell RF (1995). The effect of red and white wines on nonheme-iron absorption in humans. Am J Clin Nutr.

[10] Hurrell RF, Reddy M, Cook JD (1999). Inhibition of non-haem iron absorption in man by polyphenolic-containing beverages. Br J Nutr.

[11] Samman S, Sandström B, Toft MB, Bukhave K, Jensen M, Sørensen SS, Hansen M (2001). Green tea or rosemary extract added to foods reduces nonheme-iron absorption. Am J Clin Nutr.

[12] Kim E, Ham S, Shigenaga MK, Han O (2008). The inhibiting bioactive dietary polyphenolic compounds reduce nonheme iron transport across human intestinal cell monolayers. J Nutr.

[13] Kim EY, Ham S, Bradke D, Ma Q, Han O (2011). Ascorbic acid offsets the inhibitory effect of bioactive dietary polyphenolic compounds on transepithelial iron transport in Caco-2 intestinal cells. J Nutr.

[14] Lesjak M, Hoque R, Balesaria S, Skinner V, Debnam ES, Srai SK, Sharp PA (2014). Quercetin inhibits intestinal iron absorption and ferroportin transporter expression in vivo and in vitro. PLoS One.

[15] Hart JJ, Tako E, Kochian LV, Glahn RP (2015). Identification of black bean (*Phaseolus vulgaris* L.) polyphenols that inhibit and promote iron uptake by Caco-2 cells. J Agric Food Chem.

[16] Hart JJ, Tako E, Glahn RP (2017). Characterization of polyphenol effects on inhibition and promotion of iron uptake by Caco‑2 cells. J Agric Food Chem.

[17] Zhen AW, Nguyen NH, Gibert Y, Motola S, Buckett P, Wessling-Resnick M, Fraenkel E, Fraenkel PG (2013). The small molecule, genistein, increases hepcidin expression in human hepatocytes. Hepatology.

[18] Bayele HK, Balesaria S, Srai SKS (2015). Phytoestrogens modulate hepcidin expression by Nrf2: implications for dietary control of iron absorption. Free Radic Biol Med.

[19] Mu M, An P, Wu Q, Shen X, Shao D, Wang H, Zhang Y, Zhang S, Yao H, Min J, Wang F (2016). The dietary flavonoid myricetin regulates iron homeostasis by suppressing hepcidin expression. J Nutr Biochem.

[20] Leopoldini M, Russo N, Chiodo S, Toscano M (2006). Iron chelation by the powerful antioxidant flavonoid quercetin. J Agric Food Chem.

[21] Olthof MR, Hollman PCH, Buijsman MNCP, Amelsvoort JMM, Katan MB (2003). Chlorogenic acid, quercetin-3-rutinoside and black tea polyphenols are extensively metabolized in humans. J Nutr.

[22] Bhagwat S, Haytowitz DB, Holden JM (2014). USDA database for the flavonoid content of selected foods.

[23] Reagan-Shaw S, Nihal M, Ahmad N (2008). Dose translation from animal to human studies revisited. FASEB J.

[24] Lesjak M, Beara I, Simin N, Pintać D, Majkić T, Bekvalac K, Orčić D, Mimica-Dukić N (2018). Antioxidant and anti-inflammatory activities of quercetin and its derivatives. J Funct Foods.

[25] Torrance JD, Bothwell TH, Cook JD (1980). Tissue iron stores. Methods in hematology: iron.

[26] Moon YJ, Wang L, DiCenzo R, Morris ME (2008). Quercetin pharmacokinetics in humans. Biopharm Drug Dispos.

[27] Tang Y, Li Y, Yu H, Gao C, Liu L, Chen S, Xing M, Liu L, Yao P (2014). Quercetin prevents ethanol-induced iron overload by regulating hepcidin through the BMP6/SMAD4 signaling pathway. J Nutr Biochem.

[28] Zhang Y, Gao Z, Liu J, Xu Z (2011). Protective effects of baicalin and quercetin on an iron-overloaded mouse: comparison of liver, kidney and heart tissues. Nat Prod Res.

[29] Vanhees K, Godschalk RW, Sanders A, vanvan WaalwijkDoorn-Khosrovani SB, van Schooten FJ (2011). Maternal quercetin intake during pregnancy results in an adapted iron homeostasis at adulthood. Toxicology.

[30] Laftah AH, Ramesh B, Simpson RJ, Solanky N, Bahram S, Schümann K, Debnam ES, Srai SK (2004). Effect of hepcidin on intestinal iron absorption in mice. Blood.

[31] Mena NP, Esparza A, Tapia V, Valdés P, Núñez MT (2008). Hepcidin inhibits apical iron uptake in intestinal cells. Am J Physiol Gastrointest Liver Physiol.

[32] Frazer DM, Inglis HR, Wilkins SJ, Millard KN, Steele TM, McLaren GD, McKie AT, Vulpe CD, Anderson GJ (2004). Delayed hepcidin response explains the lag period in iron absorption following a stimulus to increase erythropoiesis. Gut.

[33] Yamaji S, Sharp P, Ramesh B, Srai SK (2004). Inhibition of iron transport across human intestinal epithelial cells by hepcidin. Blood.

[34] Chung B, Chaston T, Marks J, Srai SK, Sharp PA (2009). Hepcidin decreases iron transporter expression in vivo in mouse duodenum and spleen and in vitro in THP-1 macrophages and intestinal Caco-2 cells. J Nutr.

[35] Brasse-Lagnel C, Karim Z, Letteron P, Bekri S, Bado A, Beaumont C (2011). Intestinal DMT1 cotransporter is down-regulated by hepcidin via proteasome internalization and degradation. Gastroenterology.

[36] Muckenthaler MU, Galy B, Hentze MW (2008). Systemic iron homeostasis and the iron-responsive element/iron-regulatory protein (IRE/IRP) regulatory network. Annu Rev Nutr.

[37] Kemna E, Pickkers P, Nemeth E, van der Hoeven H, Swinkels D (2005). Time-course analysis of hepcidin, serum iron, and plasma cytokine levels in humans injected with LPS. Blood.

[38] Justino GC, Santos MR, Canário S, Borges C, Florêncio MH, Mira L (2004). Plasma quercetin metabolites: structure-antioxidant activity relationships. Arch Biochem Biophys.

[39] Lee J, Mitchell AE (2012). Pharmacokinetics of quercetin absorption from apples and onions in healthy humans. J Agric Food Chem.

[40] Patchen B, Koppe T, Cheng A, Seo YA, Wessling-Resnick M, Fraenkel PG (2016). Dietary supplementation with ipriflavone decreases hepatic iron stores in wild type mice. Blood Cells Mol Dis.

[41] Grillo AS, SantaMaria AM, Kafina MD, Cioffi AG, Huston NC, Han M, Seo YA, Yien YY, Nardone C, Menon AV, Fan J, Svoboda DC, Anderson JB, Hong JD, Nicolau BG, Subedi K, Gewirth AA, Wessling-Resnick M, Kim J, Paw BH, Burke MD (2017). Restored iron transport by a small molecule promotes absorption and hemoglobinization in animals. Science.

[42] Zhang M, Liu J, Guo W, Liu X, Liu S, Yin H (2016). Icariin regulates systemic iron metabolism by increasing hepatic hepcidin expression through Stat3 and Smad1/5/8 signaling. Int J Mol Med.

