# Supplementary material for: Quercetin inhibits intestinal non-haem iron absorption by regulating iron metabolism genes in the tissues
Source: Eur J Nutr. 2018 Mar 28;58(2):743–53. doi: 10.1007/s00394-018-1680-7 (PMC6437293; doi:10.1007/s00394-018-1680-7)
Supplement: Supplementary file 1 — Supplementary material 1 (DOC 37 KB) [file 394_2018_1680_MOESM1_ESM.doc]

**SUPPLEMENTARY MATERIAL**

**TABLES**

Table 1. Optimized LC–MS/MS parameters

| compound | retention  time  (min) | ionisation  mode | fragmentor  voltage  (V) | precursor  ion  (*m/z*) | product  ion  (*m/z*)* | collision  energy  (V)* |
| --- | --- | --- | --- | --- | --- | --- |
| quercetin-3,4’-di-*O*-glucoside | 1.46 | ni | 200 | 625 | 463, 301 | 15, 35 |
| quercetin-3-*O*-glucuronide | 2.11 | ni | 145 | 477 | 301, 151 | 20, 20 |
| isorhamnetin-3-*O*-glucoside | 2.94 | ni | 180 | 477 | 314, 243 | 30, 45 |
| quercetin | 3.65 | ni | 130 | 301 | 151 | 20 |
| genistein | 4.09 | ni | 145 | 269 | 133 | 30 |
| isorhamnetin | 4.76 | ni | 150 | 315 | 300 | 20 |

ni - negative ionization

*second number represents qualifier ion parameter
